# Supplementary figures and images for: Genetic risk for neurodegenerative conditions is linked to disease-specific microglial pathways
Source: PLoS Genet. 2025 Apr 9;21(4):e1011407. doi: 10.1371/journal.pgen.1011407 (PMC12017514; doi:10.1371/journal.pgen.1011407)

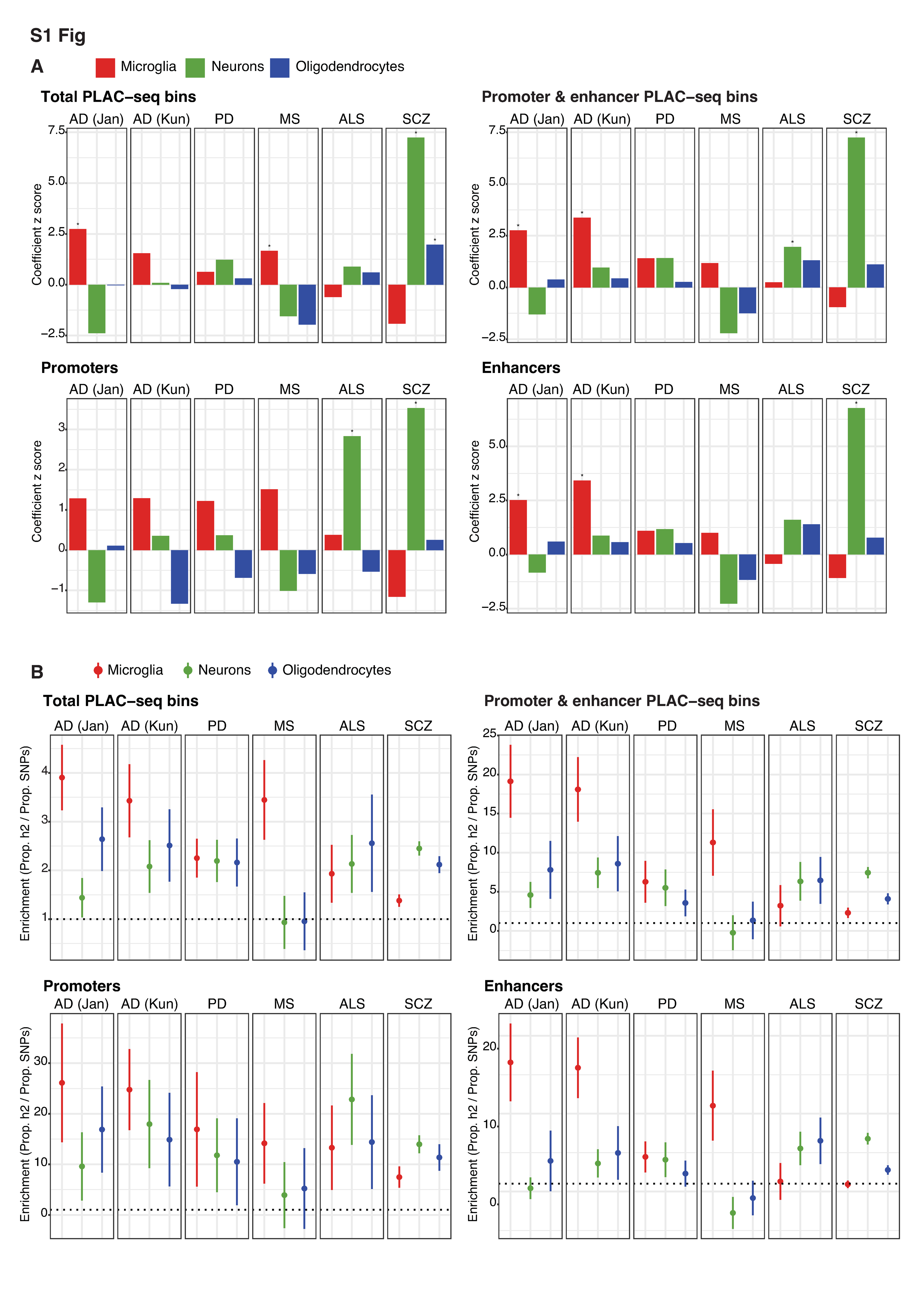

Supplement: S1 Fig — A) Partitioned heritability sLDSC coefficient z-scores for i) total PLAC-seq bins (ii) promoter and enhancer PLAC-seq bins; iii) all promoters and iv) all enhancers for microglia, neurons and oligodendrocytes in AD, PD (excluding 23andMe), MS, ALS, and schizophrenia. *transformed coefficient p-values < 0.05. B) Partitioned heritability sLDSC enrichment values defined as the ratio of the proportion of heritability to the number of SNPs (Prop. h2/ Prop. SNPs) for i) total PLAC-seq bins (ii) promoter and enhancer PLAC-seq bins; iii) all promoters and iv) all enhancers for microglia, neurons and oligodendrocytes in AD, PD (excluding 23andMe), MS, ALS, and schizophrenia. The grey dotted line represents the cutoff for enrichment (1). Error bars represent standard error. SCZ, schizophrenia. (TIF) [file pgen.1011407.s001.tif]

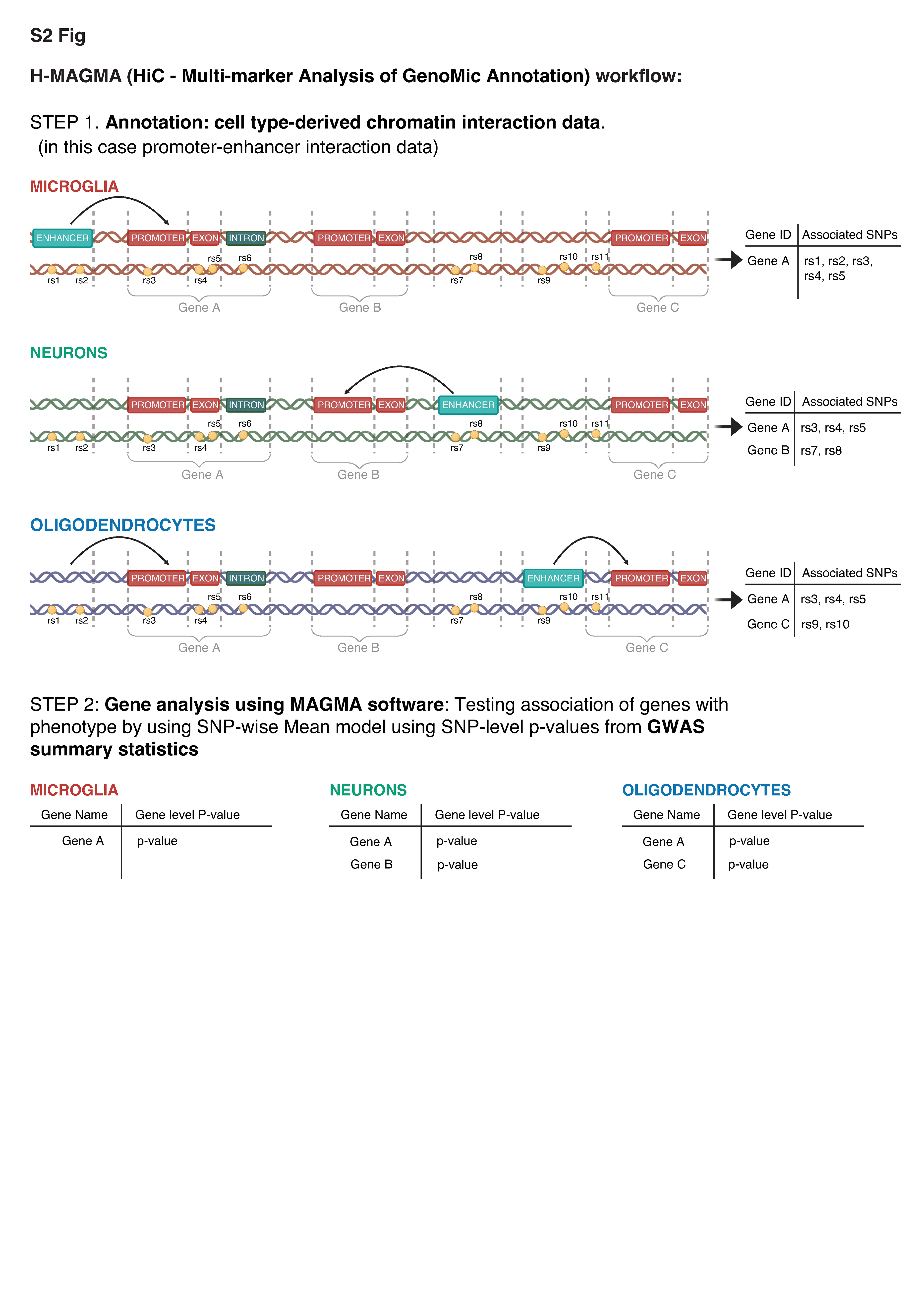

Supplement: S2 Fig — Step 1, genes are annotated to their associated SNPs using chromatin interaction data, enhancers, and promoters that were derived from each cell type. Step 2, gene analysis is performed using MAGMA software and relevant disease phenotypes (GWAS summary statistics) to identify disease-associated risk genes. (TIF) [file pgen.1011407.s002.tif]

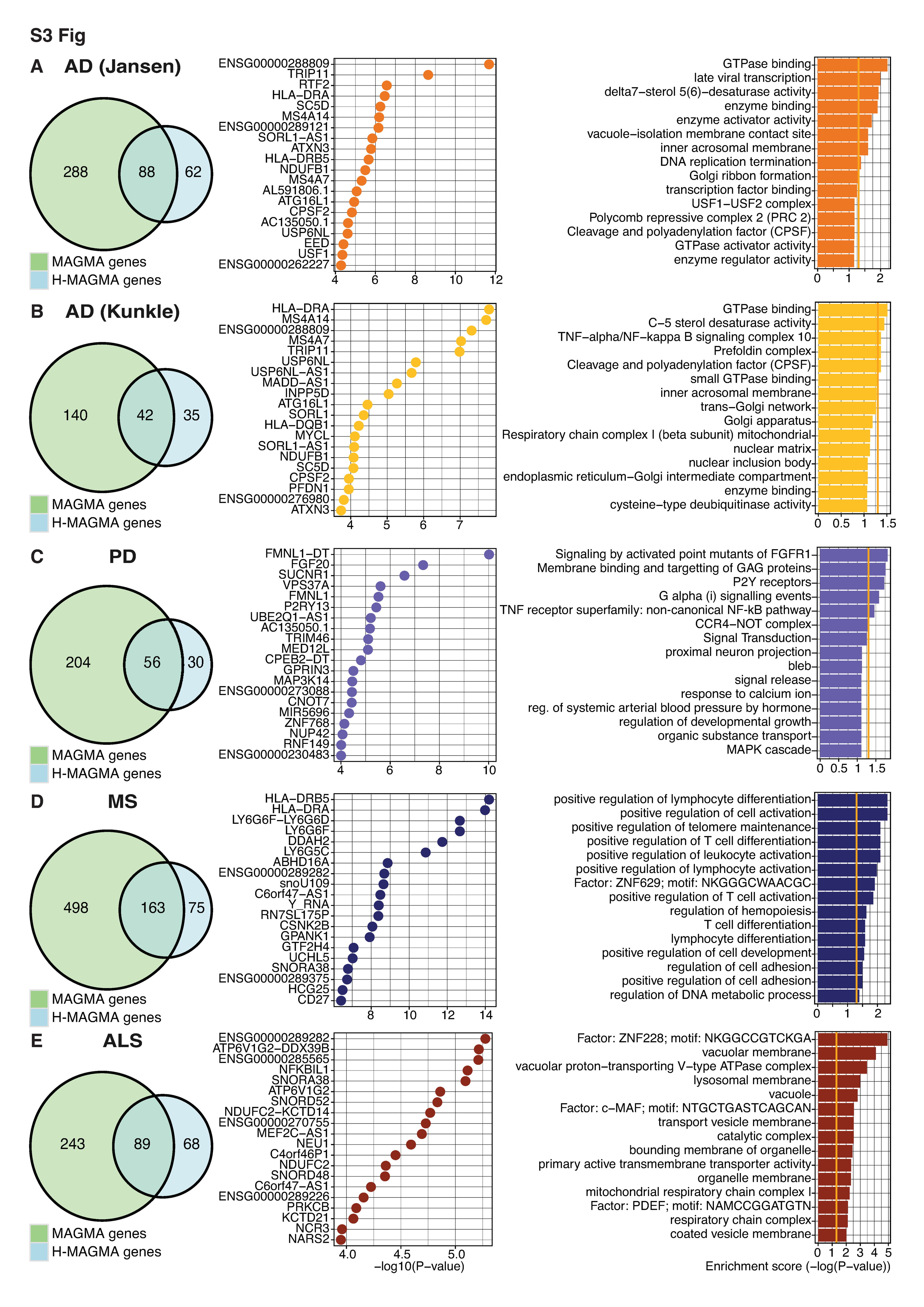

Supplement: S3 Fig — Venn diagrams (left) illustrate the overlap between genes identified by MAGMA (green) and microglia-specific H-MAGMA (blue) across diseases for: A) AD (Jansen et al. 2019) [29], B) AD (Kunkle et al., 2019)[30], C) PD (minus 23andme), D) MS, and E) ALS. Dot plots (middle) display the top 20 genes identified exclusively by H-MAGMA. Bar plots (right) represent the top pathways containing at least one of these H-MAGMA-unique genes. (TIF) [file pgen.1011407.s003.tif]

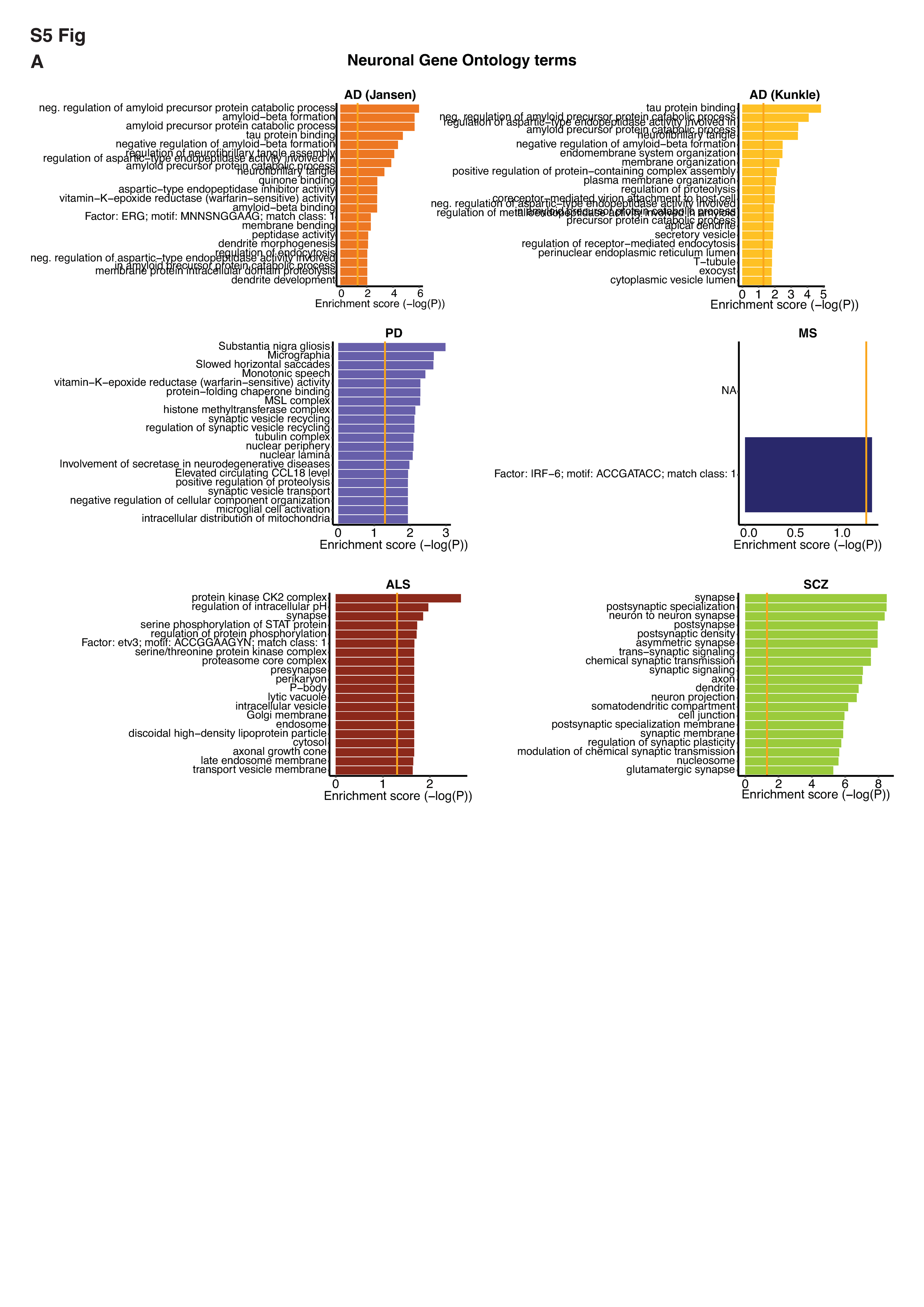

Supplement: S5 Fig — Gene ontology pathway analysis of neuronal risk genes identified by H-MAGMA for AD, PD (excluding 23andMe), MS, ALS, and schizophrenia; shown are the top 20 pathways. SCZ, schizophrenia. (TIF) [file pgen.1011407.s005.tif]

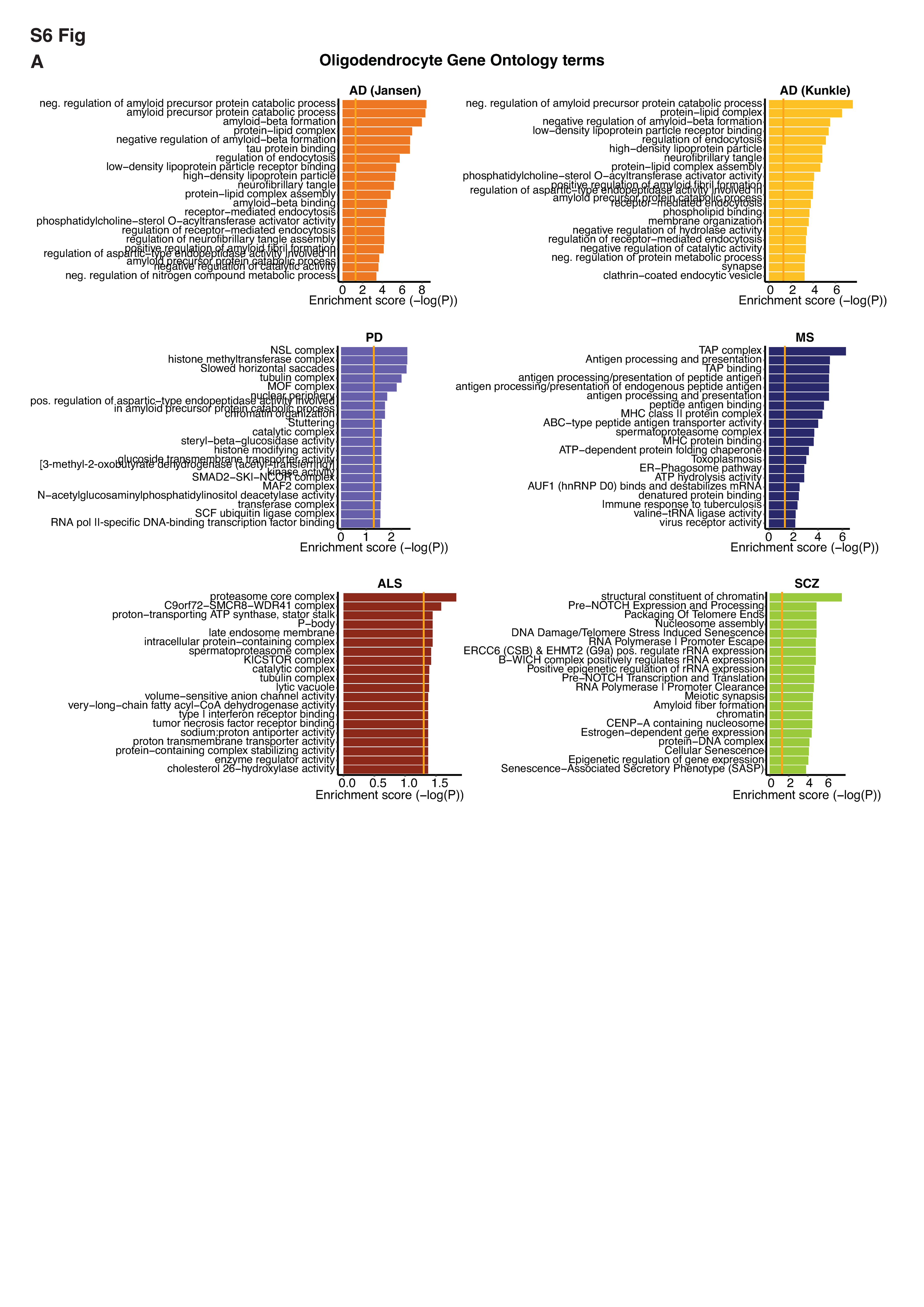

Supplement: S6 Fig — Gene ontology pathway analysis of oligodendrocyte risk genes identified by H-MAGMA for AD, PD (excluding 23andMe), MS, ALS, and schizophrenia; shown are the top 20 pathways. SCZ, schizophrenia. (TIF) [file pgen.1011407.s006.tif]

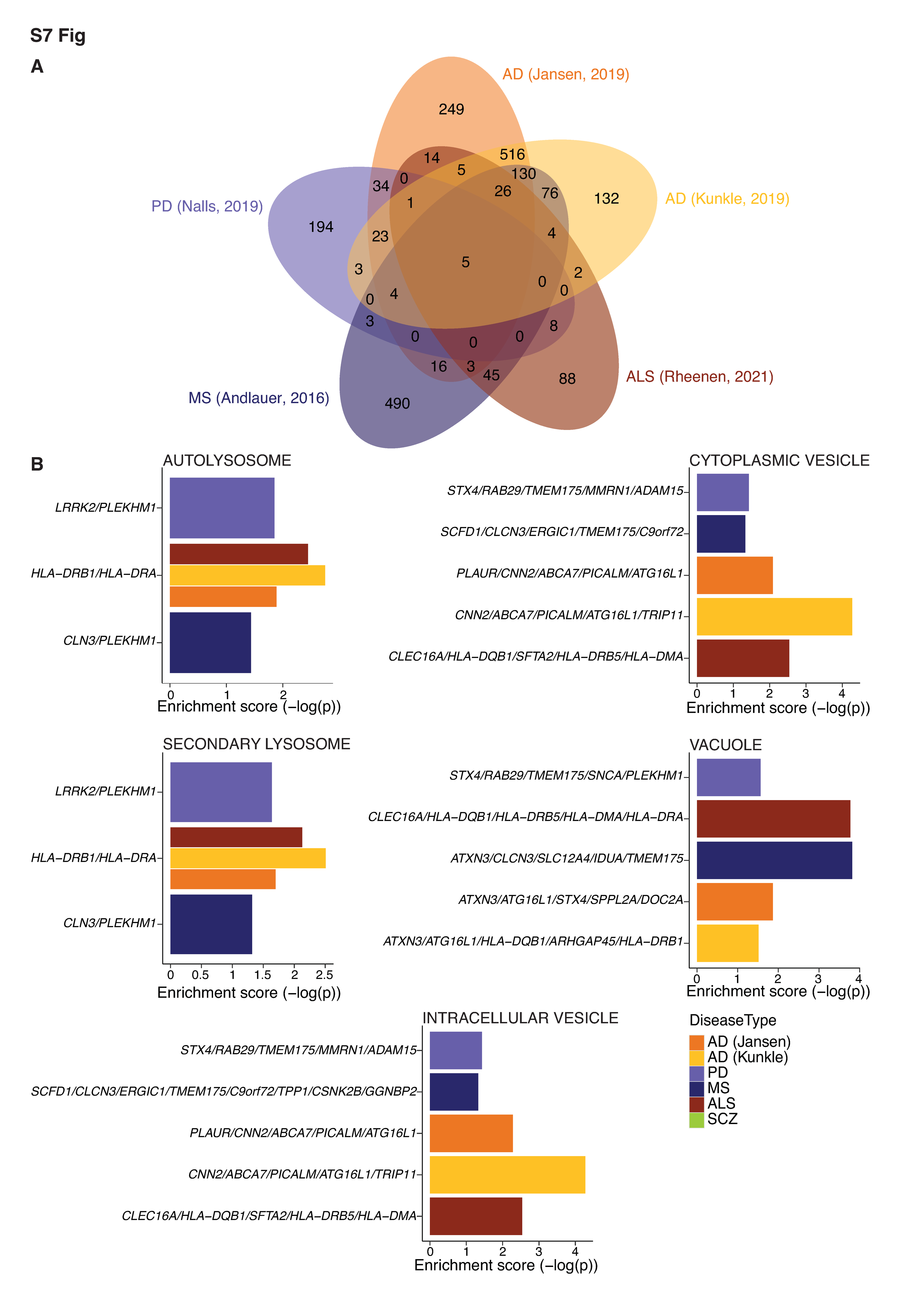

Supplement: S7 Fig — A) Number of overlapping microglial pathways across diseases. B) Bar plots of the five microglial pathways shared between AD, PD, MS, and ALS. The y-axis represents genes associated with each pathway per disease, the x-axis indicates pathway significance p-value (–log10(p-value), in relation to the disease, and colours denote different diseases. (TIF) [file pgen.1011407.s007.tif]
