# Supplementary material for: Genetic risk for neurodegenerative conditions is linked to disease-specific microglial pathways
Source: PLoS Genet. 2025 Apr 9;21(4):e1011407. doi: 10.1371/journal.pgen.1011407 (PMC12017514; doi:10.1371/journal.pgen.1011407)
Supplement: Attachment — Submitted filename: Response to Reviewers.pdf [file pgen.1011407.s015.pdf]

We thank the Reviewers for their positive and helpful comments on our manuscript. We have incorporated many of their suggestions in the revised manuscript, and we believe that they help us better convey the major findings reported in this work. Below is a point-by-point response with new data embedded and labeled according to its current place in the manuscript.

Reviewer: 1

This is an interesting manuscript, which addresses gaps in our knowledge relating to the cell-specific functions of non-coding variants identified from GWAS of neurodegenerative disorders (Alzheimer's disease, Parkinson's disease, multiple sclerosis, and amyotrophic lateral sclerosis). To address these knowledge gaps, the authors harnessed information from enhancer-to-promoter interaction maps for microglia, neurons and oligodendrocytes to identify cell type specific enrichments for these different neurodegenerative disorders. From these analyses, the authors show that while microglia were identified as the most important cell types for neurodegenerative disorders, the genes and pathways that are affected in these cells are largely unique for each of the examined disorders. While this is an interesting study, this manuscript would be strengthened by addressing the comments listed below.

We are grateful to the Reviewer for their positive critique of the manuscript as '*an interesting study*'. We have addressed the points raised by Reviewer as detailed below:

1. The abstract would benefit from including mention to the results from the analyses of the schizophrenia GWAS.

The results for schizophrenia have now been mentioned in the abstract (lines 19-27):

"*Our analysis suggests that genetic heritability for multiple neurodegenerative disorders is enriched at microglial chromatin contact sites <u>while schizophrenia heritability is predominantly enriched at chromatin contact sites in neurons followed by oligodendrocytes</u>. Through Hi-C coupled multimarker analysis of genomic annotation (H-MAGMA), we identified disease risk genes for Alzheimer's disease, Parkinson's disease, multiple sclerosis, amyotrophic lateral sclerosis and schizophrenia. We found that disease-risk genes were overrepresented in microglia compared to other brain cell types across neurodegenerative conditions <u>and within neurons for schizophrenia</u>.*"

2. P4, lines 105-115. There are a few areas in the results where there is repetition of the methods. This should be checked throughout.

The original page 4, lines 105-115, has been removed from the main text, and additional repetitive text of the methods was identified and removed throughout.

3. P5, line 122-124. Please provide the range in addition to the average distance for each cell type.

This has been addressed in the Results section (lines 129-132) as follows:

"*The average distance of these promoter-to-enhancer interactions was 200 kb for microglia (range: 3,443 – 1,003,377 bp; mean: 206,304 bp), 250 kb for neurons (2,657 – 1,008,329 bp; mean: 254,016 bp) and 150 kb for oligodendrocytes (range: 2,974 – 999,211 bp; mean: 159,230 bp) (Fig. 1c).*"

4. P6, line 162. The "Microglial chromatin interactions identify disease risk genes across multiple neurodegenerative conditions" section should include a comparison of how many disease genes would be identified by using standard gene mapping strategies that don't consider cell-specific interactions with distal gene regulatory regions. How many more genes are identified using the PLAC-seq data that would not have been identified as disease genes using standard methods? What are these genes?

We have provided lists of risk genes identified using the standard gene mapping strategy MAGMA with default 10 kb regions flanking all genes and GWAS for neurodegenerative conditions and schizophrenia (Supplemental Table 3). In addition, we have provided a subset of risk genes identified by H-MAGMA but not MAGMA (Supplemental Table 4) and the gene ontology analysis of risk genes identified by H-MAGMA only (Supplemental Table 5) for the three cell types.

Genes identified by H-MAGMA only have been mentioned in the 'Microglial chromatin interactions identify disease risk genes across multiple neurodegenerative conditions' section (lines 214-225) and in Supplementary Fig. 3.

"*Standard gene mapping approaches such as MAGMA rely on the proximity of GWAS risk variants to genes (53). The number of genes identified by assigning variants located within 10 kb of a gene using MAGMA was 2 to 3-fold higher than the number of H-MAGMA microglia risk genes identified across the neurodegenerative conditions (Supplemental Fig. 3, Supplementary Table 3). The higher number of MAGMA risk genes likely reflects the lack of cell type specificity that is provided by incorporating microglia promoter-to-enhancer interactomes. However, many H-MAGMA risk genes were not identified by MAGMA (AD Jansen, 62; AD Kunkle, 32; PD, 30; MS, 75; ALS 68 genes) (Supplemental Fig. 3, Supplementary Tables 4, 5), including the autophagy related gene ATG16L1 at the INPP5D AD locus and purinergic receptor gene P2RY13 located at the PD MED12L locus (Fig. 2c). These more distal H-MAGMA risk genes were not identified using conventional proximity-based strategies for annotating variants to genes.*"

Supplemental figure 3

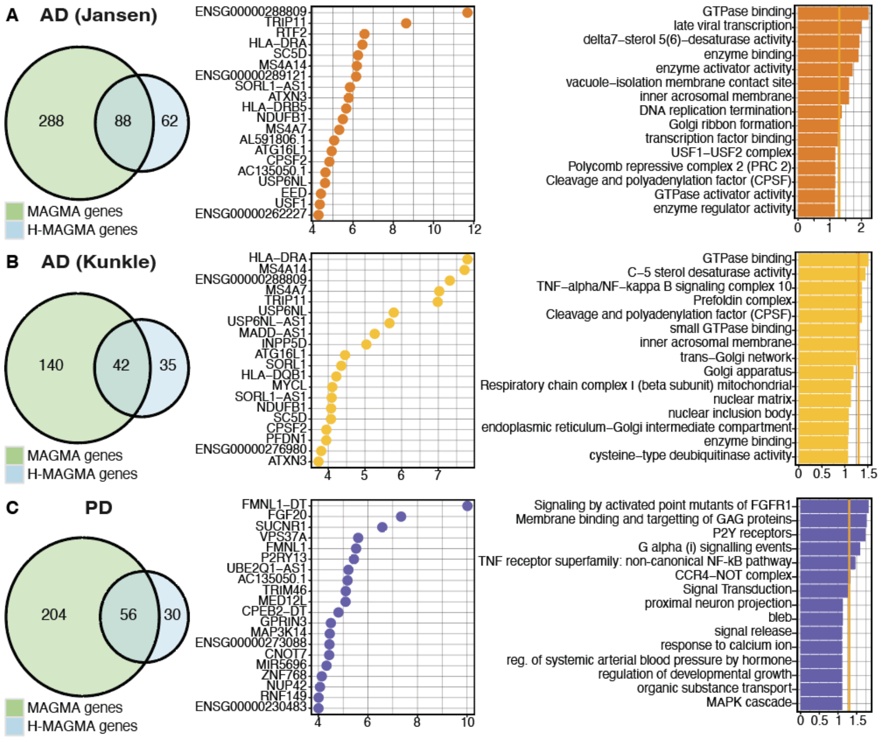

Further discussion of genes identified using H-MAGMA only (and not with standard gene mapping strategies) has been provided in the discussion:

“***H-MAGMA also identified genes located more distal from AD GWAS variants that were not found using standard mapping strategies, including endosome/endocytosis-associated genes (USP6NL, CNN2, RIN3, RAB8B), membrane-associated genes (SPPL2A, STX4, TRIP11), gene regulation (EED) and protein homeostasis (ATXN3)*.”**

And “*Inclusion of interactome data identified more distal PD H-MAGMA-risk genes in microglia , including P2RY12 and P2RY13 (immune homeostasis, mobility) (69) and UBE2Q1 (stress response, cell death) (91). H-MAGMA also highlighted FGF20, which supports dopamine secretion and protects nigral cells in PD models (92, 93), highlighting the importance of microglial enhancers for these genes in PD*.”

5. P7, lines 192-201. It would be helpful to provide numbers for the disease risk genes in the main text so readers don’t need to refer to Supplementary data.

The number of disease risk genes has been provided in the section ‘Microglial chromatin interactions are enriched for genetic risk variants associated with neurodegenerative disorders’ (lines 234-239):

“*For AD, PD and MS, the number of disease-risk genes identified using microglial enhancer contact sites (100, 64, 179 genes respectively) was comparable to the number of disease-risk genes identified using microglial promoter contact sites (111, 62, 175 genes respectively) (Supplemental Fig. 4). In ALS, more disease-risk genes were identified using microglia promoter contact sites (124 genes) compared to enhancer contact sites (47 genes) (Supplemental Fig. 4).*”

6. P8, line 216-220. This text would fit better in the discussion.

This text has now been moved to the “Discussion” (lines 347-351):

“*Disease risk genes identified in microglia across neurological conditions exhibited a microglia-specific expression profile, whereas neuron- and oligodendrocyte-associated genes lacked consistent cell-type specificity (Fig. 2d). This may reflect the distinct developmental origin of microglia from yolk sac progenitors, unlike neurons and oligodendrocytes, which arise from neural progenitor cells within the brain (71, 72, 73).*”

7. Figure 2. Use the same scales for the graphs of neurodegenerative disorders to allow for easier comparison between the diseases. Check this throughout. Also some comment should be made on why there are so many more disease genes identified for schizophrenia and how this may impact other results reported on in the paper.

The plots in Figure 2 (below) and Supplementary Figure 3 have been replaced to have the same scale across the diseases. We provided the following comment regarding why more risk genes were identified for schizophrenia (lines 195-200):

“Schizophrenia had the highest number of H-MAGMA risk genes across all cell types compared to neurodegenerative disorders. The number of identified risk genes is partially influenced by the number of associated risk variants reported in the corresponding GWAS. Schizophrenia had the most variants with a GWAS p-value below 5×10^{-8} (19,898 SNPs), more than five times the number reported for the second-highest GWAS analysed in this study (3,555 SNPs in MS).”

Figure 2

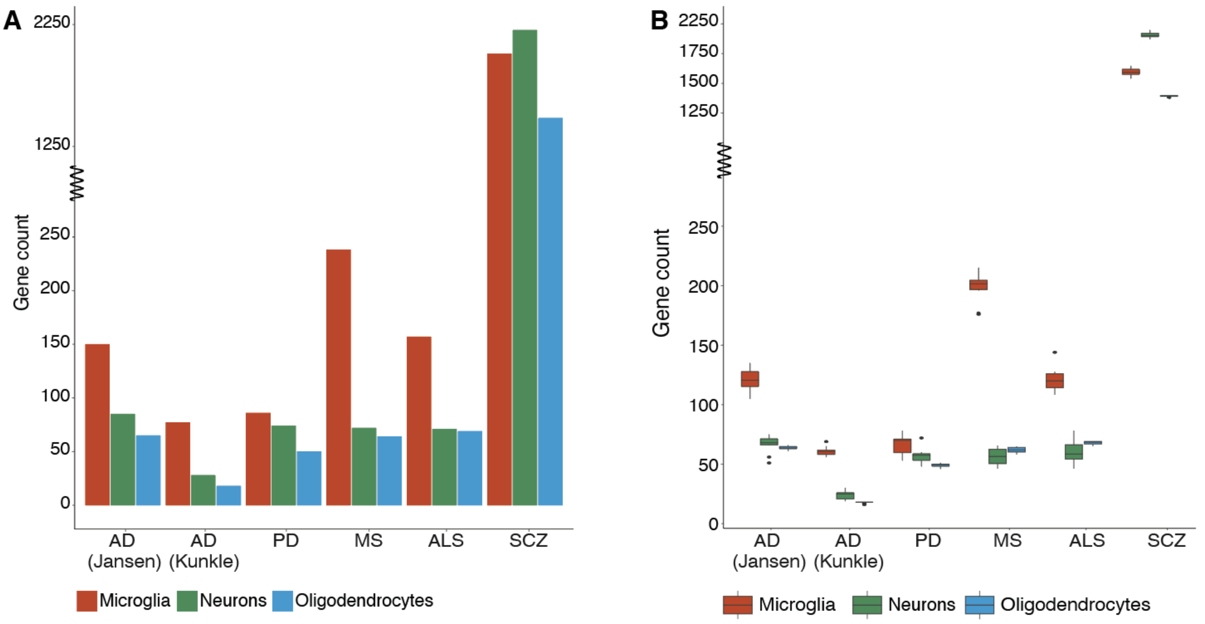

8. Figure 3B is very difficult to read. Gene names should be provided in a consistent format.

Genes shown with ensemble IDs (ENSG) instead of gene names are novel transcripts representing lncRNAs, pseudogenes and protein-coding genes. We have kept these within our figure since novel transcripts could play a crucial role in disease. However, we have added a column "gene_description" to Supplementary Tables 1,3, 4 and 5) using descriptions provided by the Ensembl gene database (including for genes ensemble IDs). Genes that need to be experimentally confirmed have been indicated in Supplemental Tables as 'TEC'.

	A	B	C	D	E	F	G	H	I	J	K	L	M	N
1	gene_ensg	gene_nam	gene_chr	gene_start	gene_end	gene_strai	nsnps	gene_zstat	gene_p	gene_fdr	gene_biot	gene_description	cell	disease
2	ENSG0000001	BIN1	2	127805603	127864864	-	304	8.28	6.1612E-17	5.7293E-13	protein_codir	bridging integrator 1 [Source:HGN	Microglia_int	AD_Jansen2019
3	ENSG0000002	APOC4-APOC	19	45445495	45452822	+	16	7.9817	7.2164E-16	3.3553E-12	protein_codir	APOC4-APOC2 readthrough (NMD	Microglia_int	AD_Jansen2019
4	ENSG0000007	PICALM	11	85668218	85780924	-	344	7.9261	1.1306E-15	3.5045E-12	protein_codir	phosphatidylinositol binding clath	Microglia_int	AD_Jansen2019
5	ENSG0000002	APOC4	19	45445494	45448753	+	10	7.8189	2.6645E-15	5.2653E-12	protein_codir	apolipoprotein C4 [Source:HGNC S	Microglia_int	AD_Jansen2019
6	ENSG0000001	ZNF296	19	45574758	45579736	-	6	7.8113	2.8311E-15	5.2653E-12	protein_codir	zinc finger protein 296 [Source:HG	Microglia_int	AD_Jansen2019
7	ENSG0000001	RELB	19	45504719	45541450	+	36	7.7059	6.4948E-15	1.0066E-11	protein_codir	RELB proto-oncogene, NF-kB subur	Microglia_int	AD_Jansen2019
8	ENSG0000002	APOC2	19	45449292	45452822	+	26	7.6708	8.5487E-15	1.1356E-11	protein_codir	apolipoprotein C2 [Source:HGNC S	Microglia_int	AD_Jansen2019
9	ENSG0000002	APOC1P1	19	45430053	45434760	+	22	7.6309	1.1657E-14	1.355E-11	transcribed_u	apolipoprotein C1 pseudogene 1 [S	Microglia_int	AD_Jansen2019
10	ENSG0000001	GEMIN7	19	45582453	45594776	+	9	7.6105	1.3656E-14	1.411E-11	protein_codir	gem nuclear organelle associated p	Microglia_int	AD_Jansen2019
11	ENSG0000001	CLPTM1	19	45457842	45496599	+	64	7.5956	1.5321E-14	1.4247E-11	protein_codir	CLPTM1 regulator of GABA type A r	Microglia_int	AD_Jansen2019
12	ENSG0000001	NECTIN2	19	45349432	45392480	+	62	7.3497	9.9309E-14	8.3952E-11	protein_codir	nectin cell adhesion molecule 2 [Sc	Microglia_int	AD_Jansen2019
13	ENSG0000002	ENSG0000002	19	45453301	45457264	-	21	7.0718	7.6494E-13	5.9276E-10	lncRNA	novel transcript	Microglia_int	AD_Jansen2019
14	ENSG0000002	ENSG0000002	11	85955395	85956015	-	29	6.9331	2.0591E-12	1.4729E-09	lncRNA	novel transcript, antisense to EED	Microglia_int	AD_Jansen2019
15	ENSG0000001	APOE	19	45409048	45412650	+	31	6.1094	5E-10	2.5831E-07	protein_codir	apolipoprotein E [Source:HGNC Syı	Microglia_int	AD_Jansen2019
16	ENSG0000001	TOMM40	19	45393826	45406946	+	56	6.1094	5E-10	2.5831E-07	protein_codir	translocase of outer mitochondrial	Microglia_int	AD_Jansen2019
17	ENSG0000001	APOC1	19	45417504	45422606	+	41	6.1094	5E-10	2.5831E-07	protein_codir	apolipoprotein C1 [Source:HGNC S	Microglia_int	AD_Jansen2019
18	ENSG0000002	ENSG0000002	19	45385284	45394133	-	26	6.1094	5E-10	2.5831E-07	lncRNA	novel transcript, antisense to PVRL	Microglia_int	AD_Jansen2019
19	ENSG0000002	ENSG0000002	19	45412632	45418225	+	53	6.1094	5E-10	2.5831E-07	TEC	TEC	Microglia_int	AD_Jansen2019

Reviewer Figure: Screenshot of Supplemental Table 1 with new 'gene_description' column indicated in yellow. Genes with ensemble IDs are indicated in green.

9. P10, line 271-273. Comparison to results obtained not making use of these interactome data would be helpful in the discussion.

We have highlighted genes that were identified using H-MAGMA that were not prioritized using a standard gene mapping strategy (MAGMA), as discussed more extensively for point 4 above.

In the discussion, we highlighted the following H-MAGMA only risk genes (lines 367-371 and 385-389):

"H-MAGMA also identified genes located more distal from AD GWAS variants that were not found using standard mapping strategies, including endosome/endocytosis-associated genes (USP6NL, CNN2, RIN3, RAB8B), membrane-associated genes (SPPL2A, STX4, TRIP11), gene regulation (EED) and protein homeostasis (ATXN3)."

And "*Inclusion of interactome data identified more distal PD H-MAGMA-risk genes in microglia , including P2RY12 and P2RY13 (immune homeostasis, mobility) (69) and UBE2Q1 (stress response, cell death) (91). H-MAGMA also highlighted FGF20, which supports dopamine secretion and protects nigral cells in PD models (92, 93),highlighting the importance of microglial enhancers for these genes in PD.*"

10. The discussion of the disease genes that were identified should be shortened and focus on genes that have not been identified through previous GWAS fine-mapping/annotation analyses i.e., those that were uncovered through the inclusion of PLAC-seq data as this is the major focus of this study. What novel information has this study provided in terms of the identification of disease genes.

We appreciate that the discussion could have delineated genes that were previously identified from those that were specifically prioritised by H-MAGMA. Reviewer 3 has asked that we prioritise the discussion to genes with prior evidence in the literature to demonstrate the robustness of our approach. We think both points are valid and have restructured parts of the manuscript to reflect this. As an example, below is a discussion of microglia AD H-MAGMA risk genes including those with prior evidence and those that were specifically annotated by H-MAGMA (underlined):

"*Microglia H-MAGMA genes previously linked to amyloid processing pathways included the ABC transporter ABCA7, vesicle-associated genes such as PICALM, BIN1 and SORL1, and protein cleavage genes such as ADAM10 and APH1B (79). PICALM and BIN1 have been further implicated to AD through microglial eQTL colocalization analyses (80) and ABCA7 and SORL1 through rare loss-of-function variants (81, 82). Further corroborating H-MAGMA in prioritizing AD risk genes, we identified genes associated with the HLA locus, immune genes (INPP5D) and microglia mobility (ADAMTS4, CASS4) that have previously been linked to AD genetic risk (28). <u>H-MAGMA also identified genes located more distal from GWAS variants that were not found using standard mapping strategies, including endosome/endocytosis-associated genes (USP6NL, CNN2, RIN3, RAB8B), membrane-associated genes (SPPL2A, STX4,TRIP11), gene regulation (EED) and protein homeostasis (ATXN3)</u>.*"

11. The discussion would benefit from some comment on why certain analyses did not uncover any significant results for certain disorders. The addition of LDSC heritability and correlation analyses would be helpful to put into context how closely the investigated disorders are related to each other.

LDSC heritability correlation analysis of neurodegenerative disorders has been published and was found to be relatively low between GWAS datasets for neurodegenerative disorders (Lona-Durazo et al. *Commun Biol* 2023). We have now commented on the relatively low genetic correlation between neurogenerative conditions in the discussion (lines 341-345):

"*A previous study using LDSC to assess genome-wide genetic correlations (rg) found a low correlation between neurodegenerative GWAS datasets, with nominally significant positive associations between AD-ALS (0.19), AD-PD (0.23), and PD-ALS (0.23) (70). This aligns with our findings that microglial regulatory regions enriched in disease are mostly disease specific.*"

12. Some discussion should be provided on why “other” interactions are so frequently observed.

The occurrence of ‘other’ interactions likely represents, in part, insulator- and repressor-associated loops. While we expect that promoters and enhancers are more functionally relevant, we cannot exclude that ‘other interactions’ may also contribute. This has now been provided as a limitation in the discussion (lines 431-436):

“*The sLDSC and H-MAGMA analysis was focused on enhancer-to-promoter interactions, given their functional significance in neurodegenerative disorders (46, 61, 62). However, PLAC-seq interactions anchored outside of promoters and enhancers (‘other interactions') potentially represent insulator- and repressor-associated chromatin loops (92, 100) that may have additional functional implications on genetic risk.*”

13. Some comment on the limitations should also be made. For example, the authors only looked at three cell types. Other cell types could also be of importance, therefore future studies would benefit from the examination of other cell types.

The limitations regarding the cell type datasets we used and the need for data on additional cell types and cell states was provided in the discussion:

Lines 438-448: “*The assignment of cell types to genetic risk and the identification of target genes depends on cell type epigenomic and chromatin interactome profiling. This has been performed for a limited number of cell types and chromatin conformation data has mostly been generated for non-dementia cases, which limited the analysis to microglia, neurons and oligodendrocytes. Recent gene expression studies have implicated vascular cell types in the genetic risk for AD (58, 101, 102). Furthermore, the expression of AD risk has been reported to be differentially enriched in microglia substates (59). These examples highlight the need for epigenomic and chromatin conformation analysis of rare cell types and substates across disease conditions using single-cell approaches.*”

Lines 333-337: “*The genetic risk of PD using single-cell gene expression analysis has identified dopaminergic neurons and oligodendrocytes as cell types that express PD risk genes (67, 68). Interestingly, PD GWAS risk was found to be enriched in microglia and monocyte chromatin accessibility regions (69), although equivalent epigenetic datasets for dopaminergic neurons are lacking.*”

We have further highlighted additional limitations of the study related to the computational tools and datasets that we used:

**Lines 415-436: “*Both sLDSC and H-MAGMA have aided in the systematic functional prediction of non-coding variants across complex traits and cell types, however, limitations of these approaches should be considered. Due to the confounding effects of linkage disequilibrium in GWAS, some of the H-MAGMA identified risk genes may result in false positives. Additionally, the directionality of expression of the identified risk genes cannot be determined but can be partially addressed by incorporating cell type eQTL data (52, 53, 98). Both H-MAGMA and sLDSC rely on the GWAS sample sizes and high SNP-heritability for accuracy of outcome (98, 99). In H-MAGMA the number of detected risk genes is dependent on the GWAS sample size and quality of chromatin interaction data. Therefore, not all identified risk genes are truly associated with the trait, necessitating functional validation. Both sLDSC and H-MAGMA were applied to European-ancestry populations due to limiting availability of large sample-sized GWAS, LD reference panel and custom genotyping arrays for other ancestries. Epigenetic and chromatin interaction data derived from pediatric resected tissue peripheral to epileptic foci was used to mitigate against aging-associated reverse*

***causality. However, epilepsy may influence cell types more broadly throughout the brain. Incorporating epigenomic datasets spanning different ages and conditions as these become available will offer deeper insights into GWAS loci that may be context specific. The sLDSC and H-MAGMA analysis was focused on enhancer-to-promoter interactions, given their functional significance in neurodegenerative disorders (46, 61, 62). However, PLAC-seq interactions anchored outside of promoters and enhancers ('other interactions') potentially represent insulator- and repressor-associated chromatin loops (92, 100) that may have additional functional implications on genetic risk.*"**

14. P26, line 501. Information is provided on age ranges, but it would also be helpful to comment on the sex and ancestry/ethnicity of these samples as all GWAS cohorts were of European ancestry. These data were also obtained from epilepsy resections. Some comment on how the use of these samples may influence results looking at other disorders.

The metadata for the PLAC-seq datasets is available from the original publication (Nott et al. *Science* 2019). This has now been summarised in the "PLAC-seq datasets" section of the Materials and Methods (lines 538-541) and in a new 'Discussion' paragraph that discusses the limitations of the study (lines 424-427):

Methods: "*PLAC-seq data for human microglia, neurons and oligodendrocytes was pre-processed by Nott et al., 2019 (40). PLAC-seq data was generated using epilepsy resections of the frontal, parietal and temporal cortex of seven individuals aged 5 months to 17 years of Caucasian, Hispanic and Pacific Islander ethnicities (6 males/1 female).*"

Discussion: "*Both sLDSC and H-MAGMA were applied to European-ancestry populations due to limiting availability of large sample-sized GWAS, LD reference panel and custom genotyping arrays for other ancestries.*"

15. P26, line 521. Information on why these specific GWAS phenotypes were selected should be included? The authors should comment on how GWAS sample sizes may impact power to uncover variants associated with the respective diseases and how this in turn may impact downstream analyses.

The justification for examining sporadic forms of neurodegenerative conditions and their associated GWAS was provided in the introduction:

"*While the genetics underlying familial cases have been informative in our understanding of disease etiology, most individuals presenting with neurogenerative disorders, including MS, have sporadic forms of the disease. Genome-wide association studies (GWAS) for sporadic neurodegenerative disorders have identified thousands of common variants associated with an increased risk of disease and highlight the heterogeneity of these disorders (27-33). GWAS risk variants generally have a relatively high prevalence in the population and exhibit smaller effect sizes, with their risk contribution believed to arise from the combined effects of multiple variants (34).*"

We have now further highlighted that sample size may impact the output of H-MAGMA in the limitations (lines 420-424):

"*Both H-MAGMA and sLDSC rely on the GWAS sample sizes and high SNP-heritability for accuracy of outcome (98, 99). In H-MAGMA the number of detected risk genes is dependent on the GWAS sample size and quality of chromatin interaction data.*

***Therefore, not all identified risk genes are truly associated with the trait, necessitating functional validation.*"**

16. P27, line 537-538. Why were different imputation quality scores applied to the different summary stats? Some information on how variant overlap between the summary statistics was assessed would also be helpful (i.e., was the number of variants included, reference populations for imputation, QC measures similar)?

We have provided further information on the quality measures that were used in the 'Quality control of GWAS summary statistics' section (lines 579-585):

"*Quality control steps included SE > 0, SNPs that have N<5 standard deviation above mean, indels were kept, chromosomes X, Y and mitochondrial chromosome were removed and multiallelic SNPs were kept. GWAS summary statistics had the following imputation quality scores: AD (Jansen 2019) >0.91; AD (Kunkle 2019) >0.4; PD >0.8; MS ≥0.8; ALS >0.95; schizophrenia >0.9. The pre-filtered imputation quality scores provided by the original GWAS were used for AD, PD, MS and ALS. For schizophrenia, we used an imputation quality score that matched the AD (Jansen 2019) GWAS.*"

17. P27, line 545. Number of SNPs included from Phase 3 1000GP should be corrected.

The text stating '22665064 million SNPs' (now line 593) has been corrected to '22,665,064 SNPs'.

18. P27, line 550. Why is the definition of promoter changed from 2,000 bp from TSS on p26, to 1,500 bp from TSS here?

PLAC-seq bins that overlap a H3K4me3 and H3K27ac peak within 2,000 bp of a transcriptional start site (TSS) used promoter annotations previously defined by Nott et al 2019. Given that the PLAC-seq loops have a resolution of 5kb, a broader definition of promoters was appropriate. To subsequently assign SNPs to promoters and exons for the annotation file used by H-MAGMA to identify risk genes it was decided that a stricter criterion would be used based on previous publications (Molineris *Mol Biol Evol.* 2011; Nair *Cell Genom.* 2024).

Molineris I, Grassi E, Ala U, Di Cunto F, Provero P. Evolution of promoter affinity for transcription factors in the human lineage. Mol Biol Evol. 2011 Aug;28(8):2173-83. doi: 10.1093/molbev/msr027. Epub 2011 Feb 18. PMID: 21335606.

Nair VD, Pincas H, Smith GR, Zaslavsky E, Ge Y, Amper MAS, Vasoya M, Chikina M, Sun Y, Raja AN, Mao W, Gay NR, Esser KA, Smith KS, Zhao B, Wiel L, Singh A, Lindholm ME, Amar D, Montgomery S, Snyder MP, Walsh MJ, Sealfon SC; MoTrPAC Study Group. Molecular adaptations in response to exercise training are associated with tissue-specific transcriptomic and epigenomic signatures. Cell Genom. 2024 Jun 12;4(6):100421. doi: 10.1016/j.xgen.2023.100421. Epub 2024 May 1. PMID: 38697122; PMCID: PMC11228891.

19. P27, line 551. Provide information on how cognate genes were defined.

We have removed the word cognate and replaced this with the Gencode gene list used for the analysis (line 616):

"*Intronic and intergenic SNPs were assigned to 'ALL' Gencode v41 genes based on cell-type chromatin interactions (see PLAC-seq datasets) with promoters and exons (52).*"

20. P27, line 540. The H-MAGMA section is difficult to follow and would benefit from the inclusion of a figure visualizing how genetic variants were mapped to target genes and how disease enrichment analyses were performed.

We have provided a schematic workflow of how genetic risk genes were identified using H-MAGMA in Supplemental Figure 2 (below), and has been indicated in the text when H-MAGMA is first mentioned in the results section (lines 189-191):

"*H-MAGMA was used to identify disease-risk genes in microglia, neurons and oligodendrocytes for AD, PD, MS, ALS and schizophrenia by incorporating PLAC-seq interactomes for the corresponding cell types (Supplemental Fig. 2).*"

Supplemental figure 2

H-MAGMA (HiC - Multi-marker Analysis of GenoMic Annotation) workflow:

STEP 1. **Annotation: cell type-derived chromatin interaction data**.
(in this case promoter-enhancer interaction data)

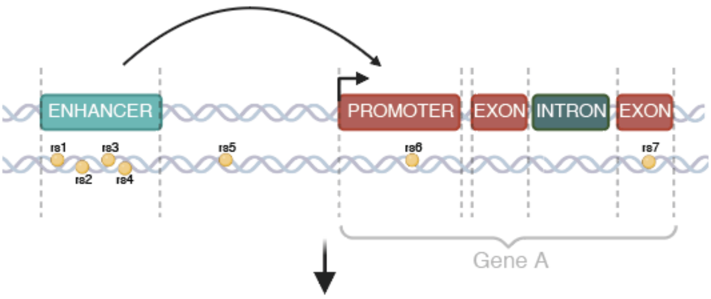

Gene Name	Associated SNPs
Gene A	rs1, rs2, rs3, rs4, rs6, rs7
Gene n	rs n, ...

STEP 2: **Gene analysis using MAGMA software**: Testing association of genes with phenotype by using SNP-wise Mean model using SNP-level p-values from **GWAS summary statistics**

Gene Name	Gene-level P-value
Gene A	p-value
Gene n	p-value

21. P29, line 604. Why were these specific mouse single cell data selected?

For the first submission, we used the Zeisel et al. (2015) single-cell RNA-seq data from mouse cortex and hypothalamus, which was the largest brain scRNA-seq dataset available and built within the EWCE package. Given limitations of translating human to mouse genomics, we have now updated this analysis using a human snRNA-seq dataset from Tsartsalis et al. (2024), which incorporates major cell types of the cortex (Fig. 2d). Additionally, in a new Fig. 2e (below), we have repeated the EWCE analysis using a human snRNA-seq dataset for microglial subtypes from Sun et al. (2023).

Lines 251-264: "*EWCE analysis revealed that the expression of microglia H-MAGMA-risk genes for all diseases was enriched predominantly in microglia, with some enrichment in lymphocytes for MS and schizophrenia (Fig. 2d). In contrast, H-MAGMA-risk genes identified in neurons and oligodendrocytes across the neurodegenerative conditions generally did not exhibit a cell type enrichment in gene expression. Only oligodendrocyte H-MAGMA risk genes identified for PD and MS showed an enrichment in oligodendrocyte signature genes (Fig. 2d). Disease risk genes identified by H-MAGMA across all three cell types for schizophrenia were characterized by matching cell type-specific gene expression (Fig. 2d). Human*

***microglia exhibit heterogeneous transcriptional profiles, a subset of which have been associated with disease conditions (59). EWCE cell type enrichment analysis indicated that microglial AD H-MAGMA risk genes were associated with inflammatory, phagocytic and cycling transcriptional profiles (Fig. 2e). In contrast, MS microglial H-MAGMA risk genes were associated with ribosome biogenesis and phagocytic transcriptional profiles and ALS with phagocytic only (Fig. 2e).*"**

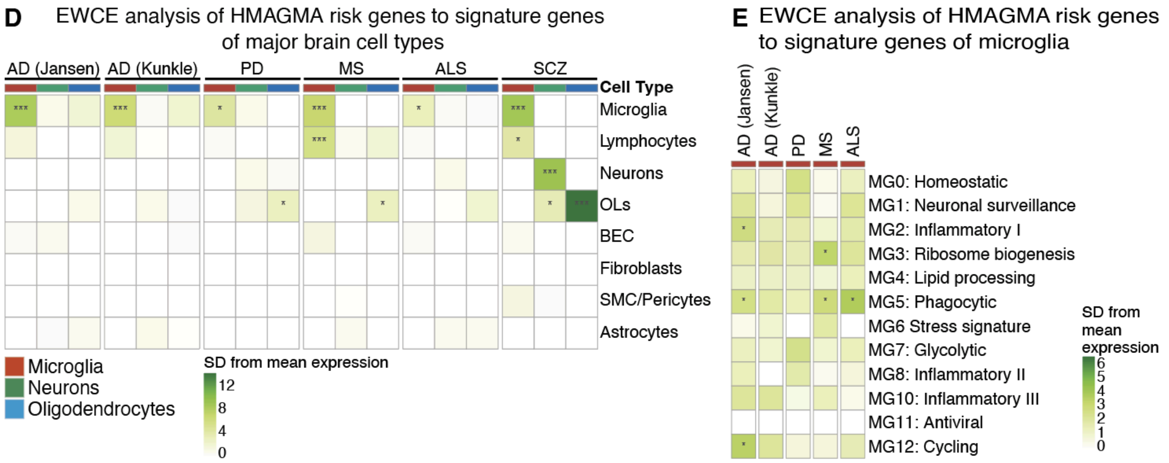

Reviewer: 2

Here the authors examined common variants (GWAS) in multiple neurodegenerative diseases (NDs). It has always been a challenge to ascribe meaning to GWAS hits as most are not in coding regions. They hypothesize that since many GWAS hits are enriched at chromatin accessibility regions that are likely enhancers or promoters that histone modifications may play a large role where cell-type specificity is at play. Hence, they form the research question of what are the target genes for these GWAS hits? And what cell-types. They use new computational tools, namely, H-MAGMA (Hi-C coupled multimarker analysis of genomic annotation) and found an enrichment of unique microglial genes in AD, PD, ALS, and MS. They used PLAC-seq to show microglia enhancer to promoter interactions were enrichment for AD, PD, and MS. Fitting with previous reports, schizophrenia was enriched in neurons and oligos. They identified some shared microglial genes across NDs, but mostly there were specific microglial genes for the separate NDs. Moreover, specific gene ontology was found across the NDs.

The paper is filled with a wealth of analysis and is very well written.

We are grateful to the Reviewer for their positive critique of the manuscript as "*filled with a wealth of analysis*" and as "*very well written*".

1. My only suggestion is to help the functional modellers out. A figure 4 should be generated to highlight the top hits (10 genes or so) in what cell types that should be functionally addressed in future studies of the NDs and schizophrenia.

A subset of the top significant genes is provided in Fig 3b. This includes H-MAGMA identified risk genes for AD, PD, MS, ALS with a H-MAGMA p<5e-8, and for schizophrenia with a H-MAGMA p<5e-12. To aid the reader, this has been more clearly signposted in the main text as follows (lines 271-274):

"*However, an intersection of GWAS-risk genes identified using H-MAGMA for microglia showed a minimal overlap between the different diseases (Fig. 3a), including for a subset of the top significant H-MAGMA risk genes (filtered on H-MAGMA p-value; AD, PD, MS, ALS p<5e-8 and schizophrenia p<5e-12) (Fig. 3b).*"

In addition, Supplementary Table 1 provides H-MAGMA risk genes listed in descending order of significance and includes a column indicating the corresponding H-MAGMA FDR corrected p-value (gene_fdr). We have now included an additional column "gene_description" to Supplementary Table 1) using descriptions provided by the Ensembl gene database (including for genes annotated with ensemble IDs).

Reviewer Figure: A screenshot from Supplemental Table 1, showing top 10 genes as ranked on FDR p-value and with the gene descriptions added (yellow):

	A	B	C	D	E	F	G	H	I	J	K	L	M	N
1	**gene_ensg**	**gene_nam**	**gene_chr**	**gene_start**	**gene_end**	**gene_stran**	**nsnps**	**gene_zstat**	**gene_p**	**gene_fdr**	**gene_bioty**	**gene_description**	**cell**	**disease**
2	ENSG0000013	BIN1	2	127805603	127864864	-	304	8.28	6.1612E-17	5.7293E-13	protein_codir	bridging integrator 1 [Source:HGN(	Microglia_int	AD_Jansen2019
3	ENSG0000022	APOC4-APOC2	19	45445495	45452822	+	16	7.9817	7.2164E-16	3.3553E-12	protein_codir	APOC4-APOC2 readthrough (NMD (	Microglia_int	AD_Jansen2019
4	ENSG0000007	PICALM	11	85668218	85780924	-	344	7.9261	1.1306E-15	3.5045E-12	protein_codir	phosphatidylinositol binding clath	Microglia_int	AD_Jansen2019
5	ENSG0000026	APOC4	19	45445494	45448753	+	10	7.8189	2.6645E-15	5.2653E-12	protein_codir	apolipoprotein C4 [Source:HGNC S	Microglia_int	AD_Jansen2019
6	ENSG0000017	ZNF296	19	45574758	45579736	-	6	7.8113	2.8311E-15	5.2653E-12	protein_codir	zinc finger protein 296 [Source:HG	Microglia_int	AD_Jansen2019
7	ENSG0000010	RELB	19	45504719	45541450	+	36	7.7059	6.4948E-15	1.0066E-11	protein_codir	RELB proto-oncogene, NF-kB subur	Microglia_int	AD_Jansen2019
8	ENSG0000023	APOC2	19	45449292	45452822	+	26	7.6708	8.5487E-15	1.1356E-11	protein_codir	apolipoprotein C2 [Source:HGNC S	Microglia_int	AD_Jansen2019
9	ENSG0000021	APOC1P1	19	45430053	45434760	+	22	7.6309	1.1657E-14	1.355E-11	transcribed_u	apolipoprotein C1 pseudogene 1 [S	Microglia_int	AD_Jansen2019
10	ENSG0000014	GEMIN7	19	45582453	45594776	+	9	7.6105	1.3656E-14	1.411E-11	protein_codir	gem nuclear organelle associated p	Microglia_int	AD_Jansen2019
11	ENSG0000010	CLPTM1	19	45457842	45496599	+	64	7.5956	1.5321E-14	1.4247E-11	protein_codir	CLPTM1 regulator of GABA type A r	Microglia_int	AD_Jansen2019

Reviewer: 3

This manuscript investigates the genetic risk for neurodegenerative conditions by leveraging enhancer-to-promoter interactomes and GWAS data to highlight microglial pathways as critical components in disease etiology. The authors employ H-MAGMA, sLDSC, and EWCE analyses to identify cell-type-specific risk genes and associated pathways. Their findings provide compelling evidence for the role of microglia in neurodegenerative diseases, with specific attention to disease-relevant pathways. The study aims to address an important topic in neurogenomics and offers valuable insights into the mechanisms underlying genetic susceptibility in neurodegenerative disorders mapping how immune processes display disease-specific patterns and identified putative cell types and genes that contribute to the genetic susceptibility of these disorders. Despite some limitations, the integration of multiple datasets and computational approaches is the greatest asset of the study. However, there are a couple of key areas where the study could benefit from some additional analyses and/or clarification to enhance its robustness and impact.

We are grateful to the Reviewer for their positive critique of the manuscript. We have addressed the points raised by Reviewer as detailed below:

1) The study would benefit from showing specific examples of the genomic regions of interest where GWAS loci overlap with PLAC-seq data. Including traces of contact frequencies or interaction loops within these regions would allow readers to better evaluate the quality and relevance of the raw data. Provide representative genome browser snapshots or visualizations (e.g., IGV or UCSC Genome Browser) highlighting GWAS regions, PLAC-seq interaction sites, and associated promoter regions. Annotating key features such as enhancer-promoter interactions, SNP locations, and nearby risk genes would make these relationships more tangible and strengthen the reader's confidence in the findings. To this reviewer, this addition would make the results more transparent and provide an intuitive way for readers to assess the data integration.

We have included UCSC browser visualization for the *INPP5D* AD locus and the *MED12L* PD locus in a new Fig. 2c (see below). Shown are tracks for H3K27ac and H3K4me3 ChIP-seq (middle tracks) and PLAC-seq interactomes (bottom track) for microglia, neurons and oligodendrocytes used for this study. We also provided the location of GWAS variants (top track). These loci included genes identified using both standard gene mapping approaches and H-MAGMA (pink), as well as more distal genes that were identified only by H-MAGMA (yellow):

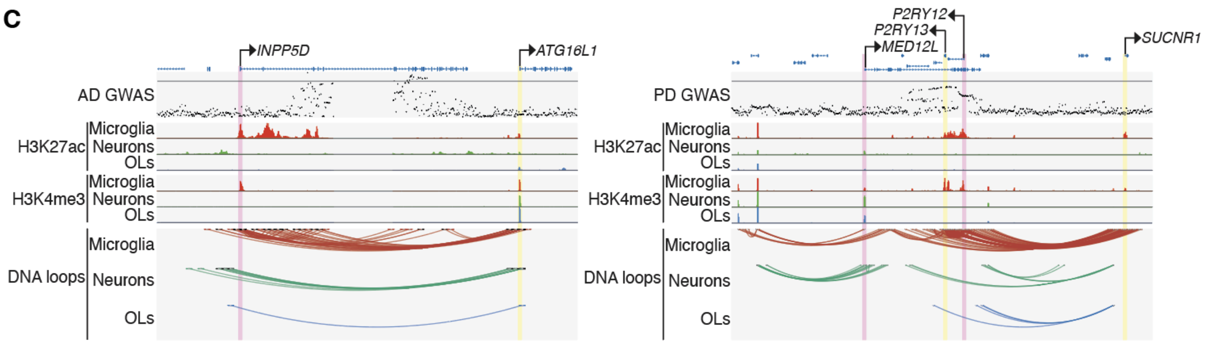

2) In regard to H-MAGMA predictions: the reliance on H-MAGMA for assigning GWAS variants to genes is a strong point of this study. However, the manuscript would benefit from additional evidence supporting the predicted gene-disease associations. Specifically, linking identified microglial genes (e.g., APOE, PICALM, and LRRK2) to the pathways implicated in the GO analysis through previously published literature or further exploration of their dataset would strengthen the claims. I would suggest authors incorporating supporting evidence from the literature or data analyses to substantiate these associations. For example, highlight prior studies that connect these genes to the identified pathways or discuss how the current dataset aligns with or diverges from known findings.

We appreciate that the discussion could have provided further evidence for H-MAGMA-risk genes with prior evidence in the literature to demonstrate the robustness of our approach. In contrast, Reviewer 1 has asked that we prioritise the discussion to new H-MAGMA-risk genes. We think both points are valid and have restructured parts of the discussion to reflect this. Below is a discussion of microglia AD and PD H-MAGMA risk genes including those with prior evidence, followed by those that were specifically annotated by H-MAGMA:

"*Pathway analysis of microglial AD H-MAGMA risk genes was associated with lipoproteins, amyloid processing, endocytosis and MHC class II. The lipid-protein complex and lipoprotein pathways included APOE, the strongest common genetic determinate of sporadic AD (77, 78). Microglia H-MAGMA genes previously linked to amyloid processing pathways included the ABC transporter ABCA7, vesicle-associated genes such as PICALM, BIN1 and SORL1, and protein cleavage genes such as ADAM10 and APH1B (79). PICALM and BIN1 have been further implicated to AD through microglial eQTL colocalization analyses (80) and ABCA7 and SORL1 through rare loss-of-function variants (81, 82). Further corroborating H-MAGMA in prioritizing AD risk genes, we identified genes associated with the HLA locus, immune genes (INPP5D) and microglia mobility (ADAMTS4, CASS4) that have previously been linked to AD genetic risk (28). H-MAGMA also identified genes located more distal from AD GWAS variants that were not found using standard mapping strategies, including endosome/endocytosis-associated genes (USP6NL, CNN2, RIN3, RAB8B), membrane-associated genes (SPPL2A, STX4,TRIP11), gene regulation (EED) and protein homeostasis (ATXN3).*

PD risk genes in microglia were associated with endo-lysosomal pathways, as previously implicated in a non-cell-type-centric manner for PD (83). These included lysosomal-associated genes (LRRK2, RAB29, PLEKHM1), membrane fusion genes (STX4, TMEM175, VPS37A, SNCA) and endocytosis (ARHGAP27). The microglia PD risk genes LRRK2, SNCA (alpha-synuclein), and TMEM175 have also been linked to rare coding mutations in PD patients (84, 85, 86). H-MAGMA-risk variants like NDUFAF2, BCKDK, and SUCNR1 are associated with mitochondrial dysfunction, with BCKDK specifically linked to Parkinsonism and Maple Syrup Urine Disease (MSUD)

***(87, 88), reinforcing evidence of mitochondrial impairment from early-onset PD mutations (PINK1, PARK7) and environmental factors (89, 90). Genes related to histone modifications were also identified as H-MAGMA-risk genes in microglia including SETD1A and FAM47E (lysine methylation), along with KAT8 and KANSL1 (lysine acetylation), which were also previously annotated as PD GWAS genes (8, 27). Inclusion of interactome data identified more distal PD H-MAGMA-risk genes in microglia , including P2RY12 and P2RY13 (immune homeostasis, mobility) (69) and UBE2Q1 (stress response, cell death) (91). H-MAGMA also highlighted FGF20, which supports dopamine secretion and protects nigral cells in PD models (92, 93), highlighting the importance of microglial enhancers for these genes in PD.*”**

3) Differentiating Disease-Specific and Shared Pathways. While the analysis highlights disease-specific pathways for microglia, neurons, and oligodendrocytes, it is unclear how much overlap exists among pathways across cell types and conditions. For instance, some pathways appear in multiple contexts but may vary in significance. The study would benefit from including a figure or table (rather than describing specific genes or pathways in the text) explicitly quantifying shared and unique pathways across diseases and cell types to improve clarity.

We have generated a new Supplemental Figure 7 showing the overlap of microglial pathways across diseases and their associated genes. The shared pathways across cell types for each disease has been provided as a new Supplemental Table 6. And a description of the shared pathways has been added to the Results section as follows (lines 310-317):

“*The only common microglial-associated GO pathways between neurodegenerative disorders were autolysosome, cytoplasmic vesicle, secondary lysosome, vacuole and intracellular vesicle (Supplementary Fig. 7). However, the combination of genes contributing to these pathways varied between diseases, with only HLA−DRB1/HLA−DRA locus shared for the autolysosomal and secondary lysosome pathways in ALS and AD (Supplementary Fig. 7). Collectively, pathway analysis confirmed the observation from gene set overlaps, indicating that microglial risk genes and associated biological pathways are mostly disease-specific.*”

Supplemental figure 7

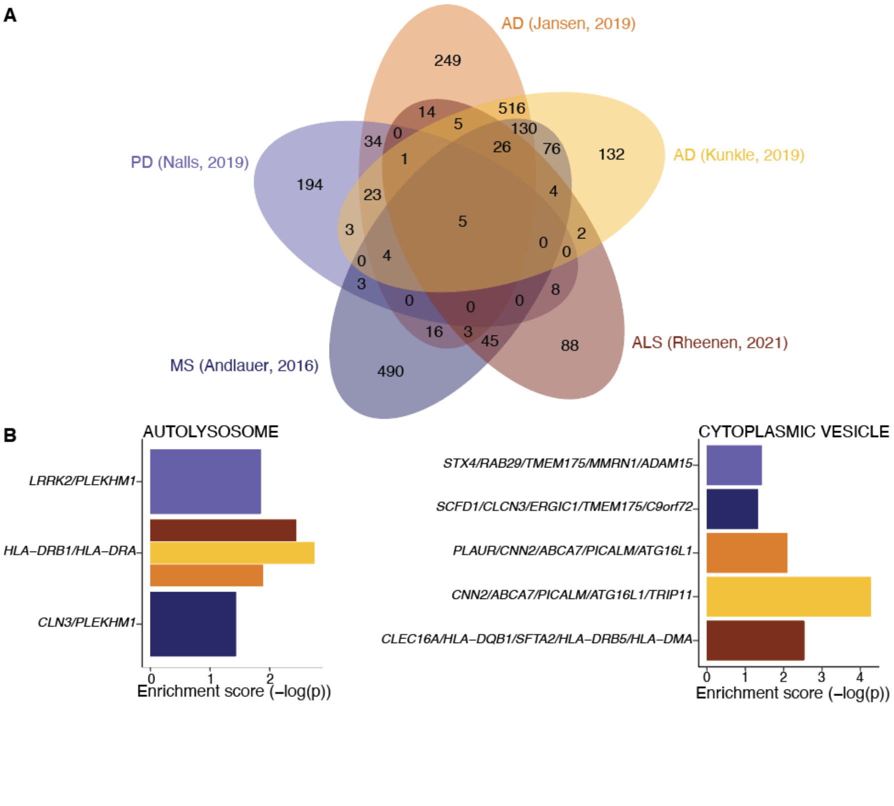

***Supplementary Figure 7 Overlap of microglial pathways across diseases and their associated genes. A) Number of overlapping microglial pathways across diseases. B) Bar plots of the five microglial pathways shared between AD, PD, MS, and ALS. The y-axis represents genes associated with each pathway per disease, the x-axis indicates pathway significance p-value (–log10(p-value), in relation to the disease, and colours denote different diseases*.**

4) Microglial Subtypes and Disease Associations: Recent studies highlight the heterogeneity of microglial subtypes in disease contexts. The authors should comment on (or even explore) how specific microglial subtypes might be linked to their findings. As a suggestion, include references to single-cell RNA-seq datasets or discuss how integrating these datasets could refine subtype-specific risk gene associations.

To examine how microglia subtypes may be impacted by H-MAGMA risk genes we performed an Expression Weighted Celltype Enrichment (EWCE) analysis using a recent snRNA-seq dataset for microglia (Sun et al. *Cell* 2023). We included a new Fig. 2e, showing AD microglia H-MAGMA-risk genes were enriched in the inflammatory, phagocytic and cycling microglial subsets:

Results (lines 261-264): *"EWCE analysis indicated that microglial AD H-MAGMA risk genes were associated with inflammatory, phagocytic and cycling transcriptional profiles (Fig. 2e). In contrast, MS microglial H-MAGMA risk genes were associated with ribosome biogenesis and phagocytic transcriptional profiles and ALS with phagocytic only (Fig. 2e).*"

Discussion (lines 352-355): *"Furthermore, AD genetic risk in microglia was most strongly associated with phagocytic, inflammatory, and cycling microglial subtypes. Consistent with this, AD pathology has been linked to impaired phagocytosis, heightened inflammatory activation, and a reduced microglial subpopulation enriched for cell cycle and DNA repair genes (74, 75, 76).*"

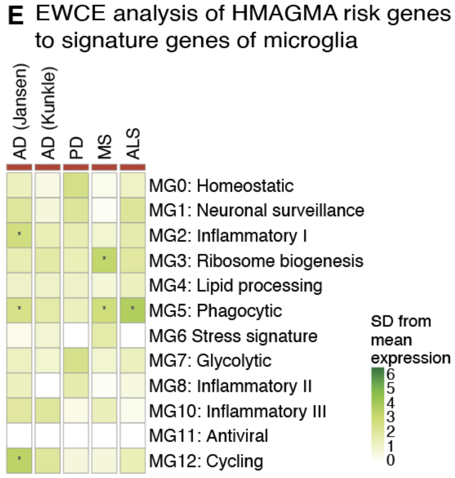

5) The study would benefit from authors touching on general limitations by:
   a. Briefly outlining any biases or assumptions inherent in using H-MAGMA or sLDSC that may impact the findings.
   b. Addressing sLDSC-Specific Limitations - noting how linkage disequilibrium patterns in GWAS studies might affect the resolution of enriched pathways or cell types, potentially leading to false positives or missed signals.
   c. Acknowledging factors of computational nature: both H-MAGMA and sLDSC rely on computational predictions, which inherently lack experimental validation and depend on the quality of input datasets (e.g., GWAS, Hi-C).

d. Highlighting any challenges in integrating results from multiple computational approaches (e.g., discrepancies between H-MAGMA and sLDSC outcomes) and how these were addressed or interpreted.

The study limitations highlighted by the review have now been added the discussion as follows (lines 415-448):

**"*Both sLDSC and H-MAGMA have aided in the systematic functional prediction of non-coding variants across complex traits and cell types, however, limitations of these approaches should be considered. Due to the confounding effects of linkage disequilibrium in GWAS, some of the H-MAGMA identified risk genes result in false positives. Additionally, the directionality of expression of the identified risk genes cannot be determined but can be partially addressed by incorporating cell type eQTL data (52, 53, 98). Both H-MAGMA and sLDSC rely on the GWAS sample sizes and high SNP-heritability for accuracy of outcome (98, 99). In H-MAGMA the number of detected risk genes is dependent on the GWAS sample size and quality of chromatin interaction data. Therefore, not all identified risk genes are truly associated with the trait, necessitating functional validation. Both sLDSC and H-MAGMA were applied to European-ancestry populations due to limiting availability of large sample-sized GWAS, LD reference panel and custom genotyping arrays for other ancestries. Epigenetic and chromatin interaction data derived from pediatric resected tissue peripheral to epileptic foci mitigate aging-related reverse causality effects. However, epilepsy may influence cell types more broadly throughout the brain. Incorporating epigenomic datasets spanning different ages and conditions as these become available will offer deeper insights into GWAS loci that may be context specific. The sLDSC and H-MAGMA analysis was focused on enhancer-to-promoter interactions, given their functional significance in neurodegenerative disorders (46, 61, 62). However, PLAC-seq interactions anchored outside of promoters and enhancers ('other interactions') potentially represent insulator- and repressor-associated chromatin loops (92, 100) that may have additional functional implications on genetic risk.*

***The assignment of cell types to genetic risk and the identification of target genes depends on cell type epigenomic and chromatin interactome profiling. This has been performed for a limited number of cell types and chromatin conformation data has mostly been generated for non-dementia cases, which limited the analysis to microglia, neurons and oligodendrocytes. Recent gene expression studies have implicated vascular cell types in the genetic risk for AD (58, 101, 102). Furthermore, the expression of AD risk has been reported to be differentially enriched in microglia substates (59). These examples highlight the need for epigenomic and chromatin conformation analysis of rare cell types and substates across disease conditions using single-cell approaches. However, our current analysis reinforces the genetic causative role of microglia in age-related brain conditions and offers biological insights into their involvement in various neurodegenerative disorders.*"**

6) Data Availability and Reproducibility: The authors provide links to code and processed data? But these links did not work for this reviewer. This issue is likely due to placeholder uploads or disruptions during the editorial process. However, as of this review, it remains unclear whether raw data or intermediate results (e.g., interaction matrices) are or will be readily accessible. Clear data availability is critical for ensuring reproducibility. Please verify that all datasets and scripts are accessible and include a more detailed description of the analysis pipeline to support transparency and reproducibility.

The GitHub repository with all the datasets and scripts for the analysis has now been made public. The code is accessible here: https://github.com/aydanasg/cell_hmagma

Conclusion

This manuscript aids in understanding the genetic underpinnings of neurodegenerative disorders by integrating genomic tools and datasets. While the findings are robust and timely, additional analyses and clarifications as highlighted above would significantly enhance the impact of the study. With these revisions, this work could make a valuable contribution to the field.

Again, we thank the Reviewer for the positive comment that this work will make "*a valuable contribution to the field*".
