# Supplementary material for: Genetic risk for neurodegenerative conditions is linked to disease-specific microglial pathways
Source: PLoS Genet. 2025 Apr 9;21(4):e1011407. doi: 10.1371/journal.pgen.1011407 (PMC12017514; doi:10.1371/journal.pgen.1011407)
Supplement: Attachment — Submitted filename: Response_to_Reviewers_auresp_2.pdf [file pgen.1011407.s016.pdf]

We thank the Reviewers for their positive comments on our revised manuscript. We have incorporated the subsequent minor comments from Reviewer 1. Below is a point-by-point response (see blue text for responses).

Reviewer: 1

The authors have carefully addressed the reviewers comments, which has strengthened the manuscript. There are only a few minor additions that are suggested as follows:

1. **Original Reviewer comment and author response**: 16. P27, line 537-538. Why were different imputation quality scores applied to the different summary stats? Some information on how variant overlap between the summary statistics was assessed would also be helpful (i.e., was the number of variants included, reference populations for imputation, QC measures similar)?

   We have provided further information on the quality measures that were used in the 'Quality control of GWAS summary statistics' section (lines 579-585): "Quality control steps included SE > 0, SNPs that have N<5 standard deviation above mean, indels were kept, chromosomes X, Y and mitochondrial chromosome were removed and multiallelic SNPs were kept. GWAS summary statistics had the following imputation quality scores: AD (Jansen 2019) >0.91; AD (Kunkle 2019) >0.4; PD >0.8; MS ≥0.8; ALS >0.95; schizophrenia >0.9. The pre-filtered imputation quality scores provided by the original GWAS were used for AD, PD, MS and ALS. For schizophrenia, we used an imputation quality score that matched the AD (Jansen 2019) GWAS.

   Reviewer response: Thank you for providing these details. As the differences in imputation quality scores could impact the results obtained, some mention of these concerns should be made in the limitations section.

   Author response: We have added the following text to the limitations in the discussion, "*Differences in imputation quality between GWAS summary statistics may introduce technical variability bias into the HMAGMA and LDSC results*".

2. **Original Reviewer comment and author response:** 18. P27, line 550. Why is the definition of promoter changed from 2,000 bp from TSS on p26, to 1,500 bp from TSS here?

   PLAC-seq bins that overlap a H3K4me3 and H3K27ac peak within 2,000 bp of a transcriptional start site (TSS) used promoter annotations previously defined by Nott et al 2019. Given that the PLAC-seq loops have a resolution of 5kb, a broader definition of promoters was appropriate. To subsequently assign SNPs to promoters and exons for the annotation file used by H-MAGMA to identify risk genes it was decided that a stricter criterion would be used based on previous publications (Molineris Mol Biol Evol. 2011; Nair Cell Genom. 2024).

   Reviewer response: Thank you for providing these details. Please add these citations to the manuscript text for clarity.

   Author response: The two citations have been added to the text (page 22).

3. **Original Reviewer comment and author response:** 20. P27, line 540. The H-MAGMA section is difficult to follow and would benefit from the inclusion of a figure visualizing

how genetic variants were mapped to target genes and how disease enrichment analyses were performed.

We have provided a schematic workflow of how genetic risk genes were identified using H-MAGMA in Supplemental Figure 2 (below), and has been indicated in the text when H-MAGMA is first mentioned in the results section (lines 189-191): "H-MAGMA was used to identify disease-risk genes in microglia, neurons and oligodendrocytes for AD, PD, MS, ALS and schizophrenia by incorporating PLAC-seq interactomes for the corresponding cell types (Supplemental Fig. 2)."

Reviewer response: Thank you for adding this figure. However, the major aspects of the methods that are difficult to follow are the different interaction data that were considered. Therefore, a figure visualizing each of the different interactions would be more beneficial.

Author response: The figure has been updated to illustrate '*each of the different interactions*'. All the interactome data was previously published and taken from the same publication using the same technique (PLAC-seq) and was described in our methods under '*PLAC-seq datasets*'. The only difference between the interactome datasets we used was the cell types, which have now been indicated in the updated figure.

Reviewer #3

The authors have responded thoroughly and thoughtfully to all critiques, making eloquent and substantive revisions. The manuscript reads well and effectively addresses prior concerns. I find the revised version to be of high quality and ready for publication.
